# Serous Ovarian Carcinoma: Detailed Analysis of Clinico-Pathological Characteristics as Prognostic Factors

**DOI:** 10.3390/cancers16213611

**Published:** 2024-10-25

**Authors:** Lamia Sabry Aboelnasr, Hannah Meehan, Srdjan Saso, Ernesto Yagüe, Mona El-Bahrawy

**Affiliations:** 1 Department of Metabolism, Digestion and Reproduction, Imperial College London, London W12 0NN, UK; l.aboelnasr22@imperial.ac.uk (L.S.A.); srdjan.saso01@imperial.ac.uk (S.S.); 2 Department of Pathology, Faculty of Medicine, Menoufia University, Shibin el Kom 6131567, Egypt; 3 Imperial College NHS Healthcare Trust, London W12 0NN, UK; hannah.meehan1@nhs.net; 4 Hammersmith Hospital, Imperial College NHS Trust, London W12 OHS, UK; 5 Division of Cancer, Imperial College London, London W12 0NN, UK; ernesto.yague@imperial.ac.uk; 6 Department of Pathology, Faculty of Medicine, University of Alexandria, Bab Sharqi 5424041, Egypt

**Keywords:** serous ovarian carcinoma, prognostic factors, histopathological parameters, tumour microenvironment, metastatic

## Abstract

Background/Objectives: Serous ovarian carcinoma (SOC) is the most common subtype of epithelial ovarian cancer, with high-grade (HGSOC) and low-grade (LGSOC) subtypes presenting distinct clinical behaviours. This study aimed to evaluate histopathologic features in SOC, correlating these with prognostic outcomes, and explore the potential clinical implications. Methods: We analysed 51 SOC cases for lymphovascular space invasion (LVSI), tumour border configuration (TBC), microvessel density (MVD), tumour budding (TB), the tumour–stroma ratio (TSR), the stromal type, tumour-infiltrating lymphocytes (TILs), and tertiary lymphoid structures (TLSs). A validation cohort of 54 SOC cases from The Cancer Genome Atlas (TCGA) was used for comparison. Results: In the discovery set, significant predictors of aggressive behaviour included LVSI, high MVD, high TB, and low TILs. These findings were validated in the validation set where the absence of TLSs, lower peritumoural TILs, immature stromal type, and low TSR were associated with worse survival outcomes. The stromal type was identified as an independent prognostic predictor in SOC across both datasets. Inter-observer variability analysis demonstrated substantial to almost perfect agreement for these features, ensuring the reproducibility of the findings. Conclusions: The histopathological evaluation of immune and stromal features, such as TILs, TLSs, TB, TSR, and stromal type, provides critical prognostic information for SOC. Incorporating these markers into routine pathological assessments could enhance risk stratification and guide treatment, offering practical utility, particularly in low-resource settings when molecular testing is not feasible.

## Simple Summary

Serous ovarian carcinoma (SOC) is the most common type of ovarian cancer, but predicting its behaviour and patient outcomes has been challenging. This research is the first to comprehensively assess histopathological features such as lymphovascular space invasion, tumour budding, the tumour–stroma ratio, the stromal type, microvessel density, tumour-infiltrating lymphocytes, and tertiary lymphoid structures in the same cohort of both primary and metastatic SOC cases. By simultaneously evaluating these features, our study provides new insights into the tumour microenvironment and its role in disease progression. This comprehensive approach highlights the prognostic value of these features and offers a straightforward method for assessing tumour aggressiveness in routine clinical practice. These findings could lead to better risk stratification and personalised treatment strategies for SOC patients, particularly in settings where access to advanced molecular testing is limited.

## 1. Introduction

Ovarian cancer (OC) is the most lethal gynaecological malignancy. It is the fifth most common cause of cancer-related deaths in women [1]. The standard first-line therapy includes surgery and platinum-based chemotherapy [2]. Although the therapeutic landscape has been enriched for OC over the years, the survival rates of OC patients have not significantly improved.

Epithelial ovarian cancer (EOC) is the most common histologic type of OC and has five main subtypes, among which serous ovarian carcinoma (SOC) is the most common subtype [3]. SOC is classified into high-grade serous ovarian carcinoma (HGSOC) and low-grade serous ovarian carcinoma (LGSOC), which have distinct differences in their molecular signatures, clinical profiles, and management protocols [3]. Given this, it is very important to clearly identify the clinicopathological features of each type and the impact of these features on tumour behaviour and survival.

An important factor known to significantly influence cancer progression is epithelial–mesenchymal transition (EMT). This first step in the metastatic cascade enables carcinoma cells to acquire mesenchymal cell features enhancing their migratory and invasive capacities, through forming tumour buds [4]. The EMT profile has also been shown to be reflected in the tumour microvascular network, which exhibits various structural and functional abnormalities [5]. Interestingly, the same factors that drive epithelial cells towards this mesenchymal phenotype also seem to drive endothelial cells towards a proangiogenic phenotype. Therefore, both tumour budding (TB) and microvessel density (MVD) have been recently considered to be morphological clues for EMT [6]. Both MVD and TB can be identified by microscopic examination of conventionally stained histological tumour sections, yet few studies have investigated the value of their evaluation as prognostic factors in SOC.

Another major determinant of tumour behaviour is the tumour microenvironment (TME), which consists of stromal myofibroblasts, inflammatory cells, and tumour vasculature [7]. Recently, growing attention has been drawn to studying the stromal characteristics of many cancer types. Stromal myofibroblasts produce different types of growth factors and signalling molecules that promote cancer progression via crosstalk with the neoplastic cells [7]. Many studies have been conducted to evaluate the ratio of tumour to stroma (TSR) and its prognostic outcome in various cancer types [8]. Other studies have proposed that tumour stroma could be subtyped according to the qualitative assessment of the maturity of collagen in the extracellular matrix [9,10]. Although these stromal features can also be easily assessed in H&E-stained tumour sections, the value of their evaluation has been addressed in a few OC studies with inconclusive results regarding their impact on tumour behaviour. Further understanding of the role of tumour stroma in OC progression may be gained by exploring its correlations with other clinico-pathological prognostic features.

The analysis of the TME in patients with a variety of solid tumours has revealed that a major subset of tumours shows evidence of a T cell-infiltrated phenotype [11]. Several studies have reported the prognostic role of tumour-infiltrating lymphocytes (TILs) in OC [12]. However, most studies have focused only on studying specific subtypes of TILs using immunohistochemistry. In addition, many scoring systems with different cut-off points were used, and few studies tested if there is a difference in the prognostic impact between the peritumoural and intratumoural TIL populations. In the same context, eosinophils, as a major element of the innate immune response, have proven to play significant roles in many solid tumours [11]. Eosinophil infiltration into tumours is referred to as tumour-associated tissue eosinophilia (TATE). Recent studies have investigated the TATE prognostic impact and its role in tumour progression, but their results were contradictory [13]. The clinicopathological value of TATE assessment in OC merits further investigation, being easy to appreciate in routine histopathology reporting.

A comprehensive literature search revealed that there has not yet been a single study that investigated all these features simultaneously in OC. Accordingly, in this study, we aimed to complete a comprehensive evaluation of haematoxylin and eosin (H&E)-stained tumour sections to characterise the tumoural epithelial and stromal histopathological features in SOC. We also aimed to assess the correlation of these features with the histological subtype, lymph node metastasis, and survival data. Although many studies have investigated the prognostic role of histopathological features in OC, little is known about changes in these features during metastatic progression. Therefore, this study also aimed to assess if there was a difference in certain tumours and the TME-related characteristics between the primary tumour site and the metastatic lesions in SOC.

## 2. Methodology

### 2.1. Sample Size and Statistical Power

The sample size for this study was determined using a power analysis conducted with G*Power software version 3.1.9.7. Based on a large effect size (f^2^ = 0.35), eight predictors (histopathological parameters), a power of 0.8, and a significance level of 0.05, it was calculated that 52 cases would be necessary to ensure adequate statistical power (80%) for detecting significant associations between the histopathological features and clinical outcomes. Consequently, our study included 51 cases in the discovery set and 54 cases in the validation set to meet this criterion.

### 2.2. Discovery Set: Analysis of Primary SOC Cases

#### 2.2.1. Included Cases

This study comprised 51 SOC cases, including 35 cases of LGSOC and 16 cases of HGSOC. All cases were of primary debulking specimens diagnosed as SOC without prior preoperative chemotherapy. Ethical approval was obtained from the Institutional Review Board of the Imperial College Healthcare NHS Trust Tissue Bank (Reference number: R10008-3A).

Patient demographics, and data including the locations of the tumours, and their macroscopic features were obtained from the original histopathology reports.

#### 2.2.2. Histopathologic Evaluation

H&E slides were retrieved from the Department of Histopathology at Imperial College Healthcare NHS Trust. The mean number of evaluated slides containing the tumoural areas from the primary tumour and metastatic site were 15 (range 6–22) and 10 (range 4–16) for each case, respectively. All slides were re-evaluated, independently from the reports, for the assessment of omental metastasis, lymph node involvement, the pathologic stage, the histological subtype, the associated non-invasive component (serous borderline tumour), psammoma bodies, necrosis, and the presence of lymphovascular space invasion (LVSI) [14].

Slides were evaluated regarding the predominant architectural pattern, tumour border configuration (TBC), MVD, TB, TSR, stromal type, TILs, tertiary lymphoid structures (TLSs), and TATE. The details of the evaluation method and scoring for each of these parameters are presented in Table 1. The histopathological assessment was conducted by two experienced histopathologists, L.S.A. and M.E., who independently evaluated each slide. To ensure a low rate of inter-observer variability, any discrepancies in scoring were resolved through a consensus discussion, with both histopathologists referring to the established criteria until agreement was achieved.

### 2.3. Validation Set: Analysis of TCGA Ovarian Serous Cystadenocarcinoma

#### 2.3.1. Data Source

This dataset comprises whole slide images (WSIs), summary data visualizations, and clinical data from a broad sampling of 617 carcinomas obtained from 600 patients, sourced from the TCGA Ovarian Serous Cystadenocarcinoma database. “https://portal.gdc.cancer.gov/projects/TCGA-OV (accessed on 24 October 2024)”.

#### 2.3.2. Access to Clinical Data

Relevant clinical data and pathology reports were retrieved using cBioPortal [24,25,26]. “https://www.cbioportal.org/study/clinicalData?id=ov_tcga (accessed on 24 October 2024)”.

#### 2.3.3. Inclusion Criteria 

Out of the 617 samples, only 106 samples labelled as “diagnostic slides” were considered for histopathological analysis. These slides contained WSIs of H&E-stained sections derived from Formalin-Fixed Paraffin-Embedded (FFPE) tissues. The remaining samples were derived from frozen sections and were not included in the analysis as frozen sections often exhibit differences in tissue architecture, staining quality, and overall morphology when compared to FFPE sections, which could introduce variability in the assessment of key histopathological parameters. By excluding frozen sections, we aimed to maintain consistency in tissue quality and morphology and enhance the comparability of results between the discovery and validation sets. All the included cases have not received prior preoperative chemotherapy. All the available WSIs were for primary ovarian sites and did not include metastatic sites.

#### 2.3.4. Evaluation Process

Digital whole slide images were downloaded from the TCGA portal and viewed using the SlideViewer 2.7 application. Following an initial examination, 54 samples (comprising 50 HGSOC and 4 LGSOC cases) were selected out of the 106 for the final analysis. Exclusion criteria included poor quality of available WSIs and/or inadequate representation of the tumour–stromal interface.

#### 2.3.5. Histopathological Assessment

Utilising the same methodology and criteria applied to the discovery dataset, each case’s available WSIs (with an average of 2 per case and a range of 1–6) underwent thorough histopathological evaluation at appropriate magnification levels, as specified in Table 1.

#### 2.3.6. Statistical Analysis

Statistical analyses were conducted using SPSS 22.0 (Statistical Package for Social Sciences; SPSS, Inc., Chicago, IL, USA). Categorical variables were analysed using the chi-square test and Fisher’s exact test (two-sided) as appropriate. A significance level of *p* < 0.05 was applied. Survival analyses included univariate Kaplan–Meier analysis followed by multivariate Cox proportional hazards regression modelling. Inter-observer variability analysis for TB, TILs, TLSs, the TSR, and the stromal type was evaluated using Cohen’s kappa (κ). Two independent pathologists (L.S.A. and M.E), blinded to each other’s assessments, scored these features in a subset of 50 randomly selected cases from both discovery and validation datasets. Kappa (K) values were generated, and agreement was reported as moderate, substantial, and almost perfect for Κ values of 0.41–0.60, 0.61–0.80, and 0.81–1, respectively [27].

## 3. Results

### 3.1. Discovery Set

#### 3.1.1. Clinicopathological Features of Included Serous Ovarian Carcinoma (SOC) Cases in the Discovery Set

**Figure 1:**
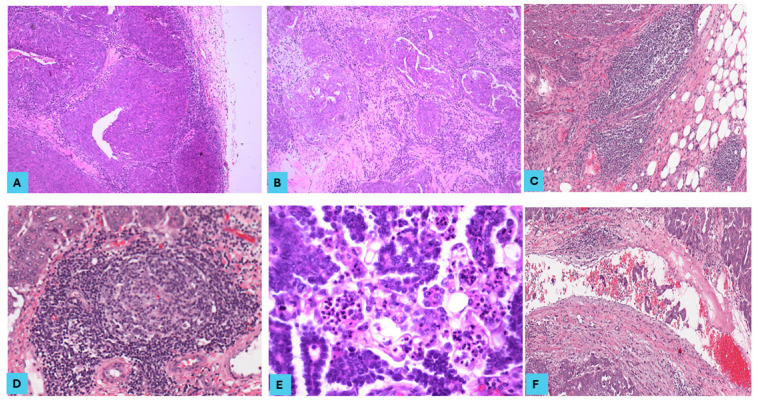
The histopathological features in high-grade serous ovarian carcinoma (HGSOC). HGSOC cases showing (**A**) a high density of peritumoural tumour-infiltrating lymphocytes (TILs) at the tumour front (H and E, ×100), (**B**) a high density of intratumoural TILs in the stroma between tumour clusters (H and E, ×100), (**C**) an immature tertiary lymphoid structure (TLS) at the invasive front of a metastatic omental lesion (H and E, ×100), (**D**) a mature TLS at the invasive front of a primary ovarian lesion (H and E, ×200), (**E**) tumour-associated tissue eosinophilia (TATE) in the centre of the tumoural component (H and E, ×400), and (**F**) Lymphovascular space invasion (LVSI) at the invasive tumour front (H and E, ×100).

**Figure 2:**
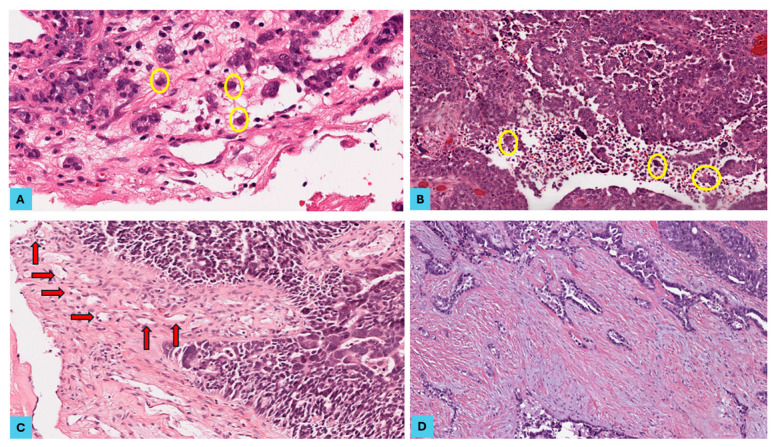
The histopathological features of epithelial–mesenchymal transition in high-grade serous ovarian carcinoma (HGSOC). HGSOC cases showing (**A**) peritumoural tumour buds (yellow circles) at the tumour front (H and E, ×400), (**B**) intratumoural tumour buds (yellow circles) in the tumour centre (H and E, ×200), (**C**) high microvessel density (MVD) (red arrows) at the invasive tumour front (H and E, ×200), and (**D**) a low tumour–stroma ratio, an intermediate stromal type with myxoid areas, and an infiltrative tumour border (H and E, ×100).

**Figure 3:**
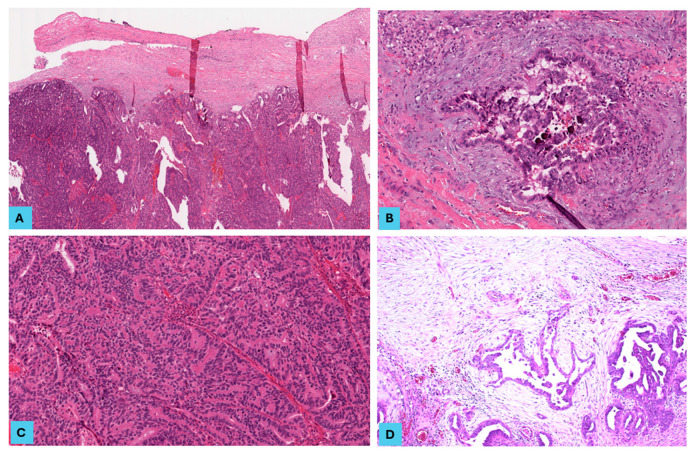
The histopathological features in primary and metastatic low-grade serous ovarian carcinoma (LGSOC). LGSOC primary lesions showing (**A**) a pushing tumour border at the tumour front (H and E, ×40), (**B**) a low density of peritumoural tumour-infiltrating lymphocytes (H and E, ×200), and (**C**) a high tumour stroma ratio (H and E, ×200). (**D**) A metastatic HGSOC lesion showing infiltrative tumour border, immature myxoid stroma, and a low density of TILs (H and E, ×100).

Most of the 51 primary SOC cases (35 cases of LGSOC and 16 cases of HGSOC) showed bilateral ovarian involvement (82.4%) with 7.2 cm as the mean of the maximum dimension of the ovarian tumour, and the same cases showed omental involvement. Lymph node (LN) samples were available for 39 cases, out of which 21 cases (53.8%) showed LN metastasis. Metastasis was detected in 40 cases (78.4%), with 3 as the mean number of metastatic lesions detected. The most frequent metastatic sites included the omentum, pelvic peritoneum, diaphragm, falciform ligament, and colon (in descending order). Only 11 cases (21.6%) were FIGO stage I, 30 cases (58.8%) were stage III, and 10 cases (19.6%) were stage IV. We then categorised tumours into two stage groups: early (stage I) in 21.6% and advanced (stage III and IV) in 78.4% of the study cohort (Table 2) (Figure 1, Figure 2 and Figure 3).

#### 3.1.2. Correlations Between Clinicopathological Features and Tumour Histological Subtype

The presence of LVSI (*p* = 0.003), high MVD (*p* = 0.0007), and high peritumoural TILs (*p* = 0.003) and the presence of TLSs (*p* < 0.0005) were associated with HGSOC. LGSOC showed a significant correlation with the micropapillary architectural pattern (*p* < 0.0005), and tumours with poor stroma (*p* = 0.029).

#### 3.1.3. Correlations Between Clinicopathological Features and Lymph Node Metastasis

The presence of LVSI (*p* = 0.0003), infiltrative TBC (*p* = 0.018), high PTB (*p* = 0.0007), high MVD (*p* = 0.002), an immature–intermediate stromal type (*p* = 0.007), and low TILs (*p* = 0.007) were associated with lymph node metastasis in both LGSOC and HGSOC cases.

#### 3.1.4. Comparison of TME Histopathologic Features Between Primary and Metastatic SOC Lesions in 40 Paired Samples

Peritumoural TIL scores in the metastatic samples were significantly lower than those in the corresponding primary tumours in both LGSOC and HGSOC cases (*p* = 0.027 and *p* = 0.009), respectively.

#### 3.1.5. Impact of the Studied Clinicopathological Features on Survival in the Discovery Set

**Figure 4:**
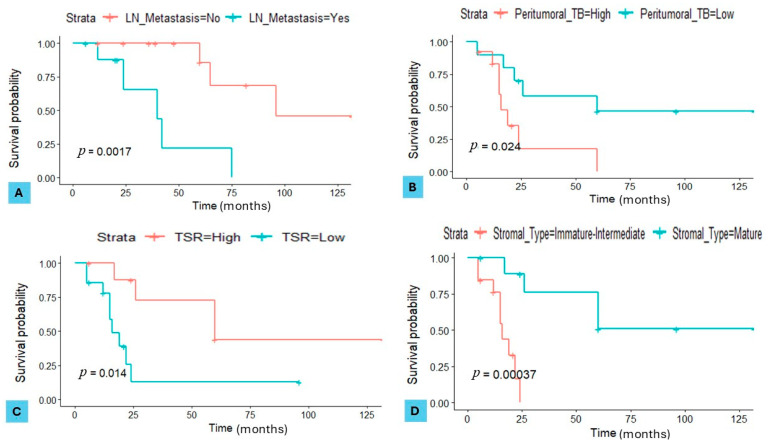
Kaplan–Meier curves for serous ovarian carcinoma (SOC) patients in the discovery set. (**A**) Overall survival (OS) stratified by the presence of lymph node (LN) metastasis (*p* = 0.0017). (**B**) Disease-free survival (DFS) stratified by peritumoural tumour budding (TB) (*p* = 0.024). (**C**) DFS stratified by tumour–stroma ratio (TSR) (*p* = 0.014). (**D**) DFS stratified by stromal type (*p* = 0.00037).

In the discovery set, the presence of lymph node metastasis demonstrated a significant impact on overall survival (OS), with a hazard ratio (HR) of 1.7 (95% CI, 1.4–2.5; *p* = 0.0017). Additionally, high PTB was significantly associated with disease-free survival (DFS), showing an HR of 2.1 (95% CI, 1.5–2.6; *p* = 0.024). A low TSR also significantly influenced DFS, with an HR of 2.3 (95% CI, 1.7–3.4; *p* = 0.014). Finally, the stromal type demonstrated a strong association with DFS, with patients having a mature stroma showing a lower risk (HR of 0.5, 95% CI, 0.2–0.9; *p* = 0.00037) compared to those with an immature–intermediate stroma (Figure 4A–D).

Multivariate Analysis of Factors Influencing Disease-Free Survival in Serous Ovarian Carcinoma (Discovery Set). The Cox proportional hazards regression analysis revealed the independent impact of the stromal type on disease-free survival (DFS) in both HGSOC and LGSOC cases in the discovery set (*p* = 0.002) (Supplementary Materials, Table S1).

### 3.2. Validation Set

#### 3.2.1. Clinicopathological Features of Included Serous Ovarian Carcinoma (SOC) Cases in the Validation Set

This study included 54 primary SOC cases (50 cases of HGSOC and 4 cases of LGSOC). Most cases showed bilateral ovarian involvement (96.3%) with 7.8 cm as the mean of the maximum dimension of ovarian tumours, and 92.6% of cases showed omental involvement. LN samples were available for 25 cases, out of which 16 cases (64%) showed LN metastasis. Metastasis was detected in 50 cases (92.6%), with 3 as the mean number of detected metastatic lesions. The most frequent metastatic sites included the omentum, pelvic peritoneum, and colon. Only 2 cases (3.7%) were FIGO stage I, 2 cases (3.7%) were stage II, 39 cases (72.2%) were stage III, and 11 cases (20.4%) were stage IV. Then, staging was categorised into 2 groups: early (stage I and II) in 7.4% and advanced (stage III and IV) in 92.6% of the study cohort (Table 2).

#### 3.2.2. Correlations Between Clinicopathological Features and Lymph Node Metastasis

The presence of LVSI (*p* = 0.017) and an immature–intermediate stromal type (*p* = 0.009) were associated with lymph node metastasis. The presence of TLSs was significantly associated with a lower rate of lymph node metastasis (*p* = 0.009).

#### 3.2.3. Impact of the Studied Clinicopathological Features on Survival in the Validation Set

**Figure 5:**
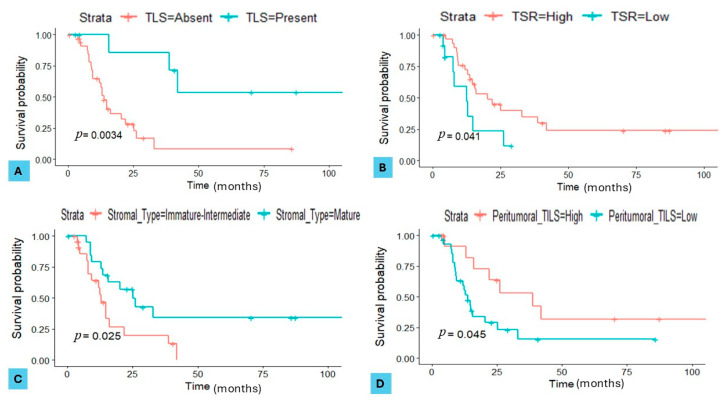
Kaplan–Meier curves for serous ovarian carcinoma (SOC) patients in the validation set. (**A**) Overall survival (OS) stratified by the presence of tertiary lymphoid structures (TLSs) (*p* = 0.0034). (**B**) Disease-free survival (DFS) stratified by the tumour–stroma ratio (TSR) (*p* = 0.041). (**C**) DFS stratified by the stromal type (*p* = 0.025). (**D**) DFS stratified by peritumoural tumour-infiltrating lymphocytes (TILs) (*p* = 0.045).

In the validation set, the presence of TLSs demonstrated a significant impact on OS, with an HR of 0.4 (95% CI, 0.1–0.8; *p* = 0.0034). Furthermore, a low TSR was significantly associated with DFS, showing an HR of 1.8 (95% CI, 0.9–2.6; *p* = 0.041). Low peritumoural TILs also significantly influenced DFS, with an HR of 2.6 (95% CI, 2.1–3.4; *p* = 0.045). Finally, the stromal type demonstrated a strong association with DFS, with patients having a mature stroma showing a lower risk (HR of 0.3, 95% CI, 0.2–0.8; *p* = 0.025) compared to those with an immature–intermediate stroma (Figure 5A–D).

Multivariate Analysis of Factors Influencing Disease-Free Survival in Serous Ovarian Carcinoma (Validation Set). The Cox proportional hazards regression analysis revealed the independent impact of the stromal type on DFS in HGSOC and LGSOC cases in the validation set (*p* = 0.029) (Table 3).

## 4. Inter-Observer Variability

The analysis demonstrated substantial agreement for TB (κ = 0.76) and the TSR (κ = 0.78) and almost perfect agreement for TILs (κ = 0.84) and TLSs (κ = 0.94) and the stromal type (κ = 0.89) between the two independent pathologists.

## 5. Discussion

Despite advances in the treatment of serous ovarian carcinoma (SOC), survival rates and therapeutic outcomes remain unsatisfactory [1,28]. Accurately identifying prognostic factors in SOC is crucial for patient risk stratification and the development of personalised therapeutic strategies, integrating standard treatments with additional therapeutic options tailored to tumour characteristics, such as antiangiogenic agents and targeted immunotherapy [29]. Our study revealed significant differences between LGSOC and HGSOC regarding features such as LVSI, MVD, and TILs. We found that lymph node metastasis correlated with several factors, including LVSI and the stromal type, and that metastatic lesions exhibited lower peritumoural TILs compared to primary tumours. Survival analysis highlighted the impact of histopathological features like lymph node metastasis and stromal characteristics on overall and disease-free survival, underscoring the prognostic value of these features in SOC. Our objective was to pinpoint histopathological parameters with a predictive value that can be readily assessed in routine clinical practice, which is particularly important in regions with limited access to comprehensive molecular testing, notably in low- and middle-income countries. By identifying histopathological parameters that can be easily adopted in routine clinical practice, we can enhance equitable access to optimised care, irrespective of the healthcare setting. This aligns with findings from comparative studies, such as that by Cobec et al., which emphasize how differences in healthcare policies and access to standardised treatment protocols based on early diagnosis and prognostic risk detection can significantly impact ovarian cancer outcomes [30].

LGSOC and HGSOC exhibit distinct biological, molecular, and clinical characteristics, with well-established cytologic and architectural features [3]. However, there has been limited investigation into the differentiation between these tumours concerning the TME and stromal features. By establishing and applying a set of definitions and criteria across two datasets including HGSOC and LGSOC cases, we found that survival analysis revealed highly consistent predictive values across both datasets, shedding light on the importance of reporting these prognostic histopathological factors in SOC, as summarised in Supplementary Materials, Table S2.

Historically, SOC has been primarily associated with spreading directly to the peritoneal cavity rather than through hematogenous or lymphatic routes [31]. However, there has been evidence suggesting that a significant portion of SOC cases may indeed involve hematogenous metastasis to sites such as the liver parenchyma and lungs [32]. The impact of the presence of LVSI in SOC had not been clearly delineated in the literature, compared to its well-defined role in other solid tumours, including breast and endometrial cancers [33,34]. According to the International Collaboration on Cancer Reporting (ICCR) and the Royal College of Pathologists of the United Kingdom (RCPath), LVSI is not among the core or the non-core items to be mentioned in the histopathology reports of SOC [35]. However, our results indicate that LVSI emerges as a critical predictor of aggressive disease in SOC, demonstrating its prognostic impact being significantly associated with HGSOC compared to LGOSC and tumours with lymph node metastasis in both subtypes, consistent with previous studies [36,37]. Although some studies suggest that the increased incidence of lymphovascular spread may be the consequence of the use of chemotherapy [38], we found that LVSI remains a significant predictor of lymph node metastasis in cases without previous chemotherapy. Thus, the detection of LVSI can serve as an indicator of lymph node involvement and may warrant lymphadenectomy, particularly in cases where imaging techniques fail to detect patients with microscopic lymph node involvement.

TBC is an important indicator of tumour invasiveness and metastatic potential [16,39]. The infiltrative pattern of TBC has been described among the invasion patterns of the metastatic high-grade serous carcinoma associated with BRCA deficiency [40]. However, its impact on tumour behaviour has not been reported. Our findings underscore a significant association between the infiltrative TBC and lymph node metastasis, indicating a more aggressive tumour phenotype. This observation aligns with previous studies highlighting the prognostic value of TBC in predicting disease progression and patient outcomes in various solid tumours, particularly colorectal cancer [16,39]. Thus, recognising the infiltrative TBC pattern in SOC should be highlighted in histopathology reports, as it is a high-risk feature that may enhance further prognostic assessments and affect patient management plans.

Angiogenesis, defined as the formation of new blood vessels, is a critical determinant in tumour progression and metastasis development [41]. MVD, an indicator of angiogenesis, is a potential prognostic factor in several malignancies, including gynaecological cancers, although the outcome of these studies is highly influenced by the choice of the antibody used [42,43,44]. Tumour blood vessels often exhibit various structural and functional abnormalities, including abnormal leakiness, facilitating the dissemination of cancerous cells into the bloodstream, and MVD could serve as a specific target in anticancer therapy [41]. Importantly, MVD can be evaluated using light microscopy and histopathological examination with or without immunohistochemistry (IHC) [17]. H&E-stained sections represent a cost-effective and readily available method for MVD assessment in all pathology units. Consistent with the existing literature, our findings demonstrate that high MVD is associated with HGSOC and tumours with lymph node metastasis, using only H&E-stained sections. This underscores that the invasive and aggressive nature of HGSOC is closely linked to its angiogenic phenotype and its capacity to establish a microvascular network.

TB emerges as another histological feature indicative of tumour aggressiveness and metastatic potential [45]. TB formation is indicative of EMT, a process widely recognised as related to cancer cell migration and a necessary step for metastasis [46]. Two forms of TB have been explored in cancer studies: intratumoural budding (ITB), found within the main tumour body, and peritumoural budding (PTB) at the invasive tumour front [16,45,47]. However, very few studies have explored TB in SOC, focusing only on ITB in HGSOC [48]. By assessing both ITB and PTB, we demonstrate a significant association between high PTB and lymph node metastasis, consistent with previous studies linking TB with advanced disease stages and a poor prognosis in various cancers [45,46]. Considering that EMT has been linked to chemoresistance in ovarian and many other cancers, this suggests that TB assessment may serve as a valuable prognostic tool in predicting disease progression and chemoresistance in SOC [49].

In the early stages of tumour invasion, tumour cells penetrate the basement membrane and stimulate stromal cells to form the TME [50]. The tumour-related stroma comprises various components such as the extracellular matrix, diverse cell types, and factors that facilitate tumour invasion and progression [50,51]. The histopathological features of the tumour–stroma interface and the TME in SOC have been documented in a limited number of studies, predominantly centred on HGSOC [7,52] identifying the TSR as a consistent and reproducible marker of the aggressive behaviour, including platinum chemoresistance [52]. Importantly, our study reveals a significant association between LGSOC and a high TSR/poor stroma, whereas HGSOC cases exhibited a low TSR/rich stroma. This underscores the potential role of the stroma in shaping the slow progression rate observed in LGSOC compared to HGSOC.

Tumour-infiltrating lymphocytes (TILs) represent a crucial element of the TME, exerting notable effects on disease progression and patient outcomes [12,20]. Our results reveal a significant correlation between elevated peritumoural TILs and HGSOC, aligning with prior research findings [53]. Disruptions in genes involved in DNA repair mechanisms lead to increased mutational burdens in HGSOC, with cases exhibiting homologous recombination deficiency (HRD) displaying a high expression of neoantigens and a T-cell-inflamed tumour phenotype [53,54]. Notably, the elevated TIL levels observed may be interconnected with other identified features associated with HGSOC in our study, such as increased MVD. Previous investigations in cholangiocarcinoma and cutaneous malignant melanoma have highlighted a positive correlation between high MVD and TILs [55,56]. Furthermore, the low TSR/high stromal percentage in HGSOC might contribute to elevated TIL levels, as ECM harbours ligands like fibronectin and laminin, providing structural attachment sites for migrating immune cells [57].

Although studies investigating immune features or immunotherapy trials in LGSOC are still limited, immune cell infiltration has been shown to be a critical parameter for assessing the immunotherapy response and a significant prognostic factor in other types of ovarian cancer, including HGSOC [58]. In particular, the presence of TILs and their interaction with immune checkpoint molecules, such as PD-1/PD-L1, play a key role in determining treatment outcomes [58,59]. Studies have shown that higher levels of PD-1+ TILs or PD-L1+ TILs are associated with better survival outcomes, suggesting that these immune markers may help predict responses to immunotherapies [58,59]. Research in HGSOC indicates that the combined analysis of PD-L1 expression and CD8+ TILs can stratify patients based on prognosis [60]. Additionally, the evolution of TIL populations during chemotherapy, particularly in the peritumoural and intratumoural regions, could further inform treatment strategies [61]. Although evidence is still emerging, these findings highlight the potential of TILs as a biomarker for guiding immunotherapy and other treatment approaches in SOC, reinforcing the need for incorporating TIL evaluation in routine reporting in OC cases.

TLSs emerge in nonlymphoid tissues during carcinogenesis [62,63]. Despite numerous studies reporting the TLSs’ positive impact on cancer prognosis and immunotherapy outcomes, their association with other clinicopathological factors, particularly in SOC remains inconclusive [22,63]. In our study, TLSs were identified in 50% and 20% of HGSOC cases in the discovery and validation cohorts, respectively. Similar findings have been recently reported in an HGSOC study focusing on TLSs [64]. Notably, and to the best of our knowledge, our study is the first to compare TLS presence between HGSOC and LGSOC. While LGSOC cases did not exhibit TLSs, a larger cohort is necessary to confirm this observation. Moreover, TLSs and peritumoural lymphocytes displayed significant associations with reduced rates of lymph node metastasis, underscoring their potential as prognostic markers in SOC. Since the cytokine and chemokine profile associated with TLS induction may determine whether TLSs inhibit or promote cancer invasiveness [65], further research is needed to investigate the cytokine profiles linked with TLSs and their associations with various metastatic behaviours.

TATE represents a component of the immune response associated with cancer whose prognostic significance is unclear [66,67]. We only observed TATE in a few cases within both the discovery and validation cohorts and thus no conclusions can be drawn on their association with clinicopathological characteristics or survival. However, peritumoural features, including PTB and peritumoural TILs, along with stromal characteristics such as the TSR and stromal type, were significant predictors of disease-free survival (DFS) in SOC. These findings highlight the importance of considering both tumour epithelial and stromal characteristics in prognostic modelling and personalised treatment approaches for SOC.

We report that the non-mature stromal type is associated with a higher rate of lymph node metastasis and poor DFS. The results of a recent study revealed that, in the early stages, cancer invasion induces a desmoplastic reaction, while in the later stages, there is degradation of the stroma, thereby facilitating tumour invasion and progression [68]. The independent impact of the stromal type on DFS was consistent in both discovery and validation cohorts. These observations are highly concordant with previous studies in rectal and lung cancer [9,69]. Thus, the stromal type has potential clinical relevance as a prognostic marker in SOC.

Prior research has demonstrated inconsistencies in the biomarker status between primary and metastatic tumours [70,71,72]. Building upon the concept of cancer immunoediting, we hypothesised that discrepancies in the immune landscape between primary and metastatic SOC tumours could similarly occur [73]. Our study represents the first investigation to explore and reveal a significant disparity in peritumoural TIL scores between primary and metastatic lesions within both LGSOC and HGSOC cohorts. We observed significantly lower TIL scores in metastatic samples compared to their corresponding primary tumours. This finding underscores the dynamic nature of the TME, reflecting an immunosuppressive TME during cancer dissemination in SOC. Further assessment of the factors contributing to this phenomenon could offer valuable insights for developing targeted therapies to control the metastatic spread in SOC.

An important aspect of our study lies in the distinct clinical outcomes and clinicopathological associations observed for the parameters under investigation, notably TB and TILs, across the intratumoural and peritumoural compartments. To the best of our knowledge, such a discrepancy has been rarely reported in SOC. Different levels of programmed cell death ligand-1 (PD-L1) expression were observed between invasive and central tumour segments in epithelial ovarian carcinoma [51]. In our study, both immature TLSs and mature TLSs were predominantly localised at invasive tumour margins as compared to the intratumoural area, consistent with previous studies [47]. This disparity signals spatial heterogeneity within the TME in SOC, emphasising the importance of a comprehensive histopathological examination.

Our findings underscore the necessity for standardised scoring criteria to ensure uniformity and reproducibility in tumour assessment. Currently, there is no available evidence indicating functional distinctions between peritumoural and intratumoural TILs. Given this knowledge gap, it has been proposed to assess TILs at the tumour’s invasive border as a distinct parameter from those in the inner stroma [20]. Accordingly, we examined all whole sections and covered numerous fields and tissue segments in our scoring methodology, subsequently aggregating average scores to account for this spatial heterogeneity. In the same context, other investigators have reported tissue microarray study limitations, as they do not account for intratumoural heterogeneity [74].

In addressing the implications of our findings, it is essential to underscore the importance of histopathological features predictive of lymph node metastasis in SOC. These features hold considerable value, particularly in cases where no LN dissection is performed, a common scenario in SOC management. Moreover, this study underscores the simplicity and practicality of our methodology, making it highly adaptable for routine adoption in clinical practice and potentially feasible for training artificial intelligence systems. The inter-observer variability results indicate a high level of reproducibility in the scoring of these histopathological features, thereby strengthening the reproducibility of these parameters’ assessment and the reliability of our findings. The straightforward scoring criteria utilised in our assessment provide a clear framework for evaluating histopathological parameters. For instance, the assessment of TBC involves distinguishing between pushing and infiltrative patterns, a task easily achievable through routine histopathological examination. Similarly, the evaluation of TB entails counting the number of buds per high-power field, along with well-defined criteria facilitated by standard microscopy techniques. Additionally, the determination of the TSR and stromal type relies on visually discernible features, such as the proportion of stromal tissue and the composition of collagen fibres, respectively. It is worth noting that the TIL scoring adhered to guidelines, specifically the recommendations of the International TILs Working Group 2014 [20], ensuring a standardised evaluation and comparability with the existing literature. By employing such straightforward scoring criteria, we have ensured a standardised approach that can be readily implemented in pathology laboratories worldwide. This simplicity not only enhances the reproducibility of our findings but also facilitates their translation into actionable insights for patient care and management, irrespective of the healthcare setting.

## 6. Conclusions

Our study identifies key histopathological features, including LVSI, TBC, TILs, and stromal characteristics (the TSR and stromal type), as significant predictors of aggressive behaviour in both LGSOC and HGSOC. The stromal type emerged as an independent prognostic marker for DFS. MVD and TB were associated with EMT-driven tumour aggressiveness and metastatic potential. These findings are highly relevant to clinical practice, particularly in low-resource settings where access to molecular testing may be limited. Routine histopathological evaluation and reporting can serve as a practical and cost-effective method for enhancing risk stratification, guiding prognosis and personalised treatment approaches without the need for advanced molecular assays. Furthermore, our study has shed light on the dynamic nature of the TME by revealing significant disparities in peritumoural TIL scores between primary and metastatic lesions within both LGSOC and HGSOC cohorts. This underscores the critical role of conducting the histological evaluation at multiple disease sites and stages in enhancing our understanding of tumour spatial and temporal progression, which is critical for developing tailored management strategies.

## Figures and Tables

**Table 1:** Criteria used to assess and classify the studied histopathological parameters.

Parameter	Method of Assessment	Categories	Corresponding Criteria
Predominant architectural pattern	All slides were assessed. We reported the most common pattern observed at sections of primary and metastatic lesions independently [15].	- Micropapillary	- Finger-like papillae with a length 5 times their width and without a fibrovascular core
		- Papillary	- Papillae with a prominent fibroconnective tissue core
		- Solid	- Sheets of tumour cells
		- Cribriform or “pseudoendometrioid”	- Tumour cells growing in a glandular pattern or nests with punched-out micro-lumens
		- Transitional cell-like	- Wide core papillae with stratified layers of tumour cells
		- SET	- Solid/pseudoendometrioid/transitional cell-like
Tumour border configuration (TBC)	All slides were assessed. We reported the most common configuration of the tumour-invasive front observed at sections of primary and metastatic lesions independently [16].	- Pushing - Infiltrative	- Well-defined and smooth borders at the interface between the tumour and surrounding tissue - Tumours that do not show a clear transition at their extension into the surrounding tissue
Microvessel density (MVD)	All slides representing the primary tumour were screened using 4× and 10× objectives to select the slide with “hot areas” where high densities of microvessels were detected at the tumour invasive front. Microvessels were counted per 10 high-power fields using a 40× objective and the mean numbers were reported [17,18].	- Low - High	- The mean count of microvessels/10HPFs ≤ median - The mean count of microvessels/10HPFs > median
Tumour budding (TB)	All slides representing the primary tumour were scanned using a 4× objective, and the slide including the most invasive tumour area was selected. TB was defined as a single tumour cell or cluster of up to 5 cells. We assessed intratumoural budding (ITB), in the tumour centre, and peritumoural budding (PTB), at the tumour front. Tumour buds were counted per 10 HPFs using a 40× objective, and the mean numbers were reported [16].	- Low - High	- The mean TB count/10HPFs < 5 - The mean TB count/10HPFs ≥ 5
Tumour–stroma ratio (TSR)	- All slides representing the primary tumour were scanned using a 4× objective, and the slide including the most invasive tumour area was selected. Then, using a 10× objective, we selected an area at the invasive tumour front where tumour cells had to be present surrounding a stromal tissue at all borders of the evaluated field [16].	- Stroma-rich - Stroma-poor	- Proportion of stroma ≥ 50% - Proportion of stroma < 50%
Stromal type	All slides representing the primary tumour were scanned using a 4× objective, and the stromal type was assessed at the most invasive frontal zone using a 40× objective lens [19].	- Mature - Intermediate - Immature	- Multiple layers of mature collagen fibres - Mixed mature collagen and immature keloid-like fibres - Only immature keloid-like fibres within the myxoid stroma
Tumour-infiltrating lymphocytes (TILs)	All slides were assessed. The average percentage of TILs was reported separately for primary and metastatic lesions. - Intratumoural TILs were defined as lymphocytes in tumour nests having cell-to-cell contact with no intervening stroma and directly interacting with carcinoma cells. Intratumoural TILs were scored using a 20× objective, according to the recommendations of the International TILs Working Group 2014, as a percentage of the area occupied by mononuclear inflammatory cells over the total intratumoural area [20]. - Peritumoural TILs were defined as TILs at the invasive margin, which extends up to 1 mm from the border separating the malignant nests from the host tissue. Peritumoural TILs were scored, according to Klintrup et al. [21] for scoring the inflammatory infiltrate at the invasive margin, and classified into four scores: 0 = no inflammation 1 = mild and patchy 2 = a band-like infiltration 3 = prominent forming a cup-like zone	- Low intratumoural TILs - High intratumoural TILs - Low peritumoural TILs - High peritumoural TILs	- Average TIL percentage ≤ 10% - Average TIL percentage > 10% - Klintrup score 0 or 1 - Klintrup score 2 or 3
Tertiary lymphoid structures (TLSs)	All slides were assessed regarding TLSs, mainly located at the invasive tumour front. - The immature TLS/lymphoid aggregate was defined as the accumulation of lymphocytes and plasma cells without a germinal centre (GC). - The mature TLS/lymphoid follicle was defined as aggregates of lymphocytes with a GC [22].	- Absent - Present (immature, mature)	
Tumour-associated tissue eosinophilia (TATE) count	- It was assessed collectively within the tumour centre (intratumoural) and in the stroma at the invasive tumour margin (peritumoural). All slides representing the primary and metastatic tumour were scanned, the slide with a high density of eosinophilic infiltration was selected to count the number of eosinophils per 10 high-power fields using a 40× objective, and the mean numbers were reported [23].	- Low eosinophil count - High eosinophil count	- Mean eosinophilic count/10 HPFs < 10/HPF - Mean eosinophilic count/10 HPFs ≥ 10/HPF

**Table 2:** Clinicopathological characteristics of studied SOC cases in the discovery set (N = 51) and the validation set (N = 54).

Variables	Discovery Set SOC Cases N (%) 51 (100)	Validation Set SOC Cases N (%) 54 (100)
Age		
Mean	55	57.5
≤mean	24 (47.1)	24 (44.4)
>mean	27 (52.9)	30 (55.6)
Laterality		
Unilateral	9 (17.6)	2 (3.7)
Bilateral	42 (82.4)	52 (96.3)
Tumour extension to the surface of the ovary		
Absent	10 (19.6)	7 (13)
Present	41 (80.4)	47 (87)
Cut section		
Solid	19 (37.3)	28 (51.9)
Solid-cystic	32 (62.7)	26 (48.1)
Primary tumour size (Max. dimension in cm)		
Mean	7.2	7.8
≤mean	25 (49)	25 (46.3)
>mean	26 (51)	29 (53.7)
Omental involvement		
Absent	9 (17.6)	4 (7.4)
Present	42 (82.4)	50 (92.6)
Lymph node (LN) metastasis		
N. Of cases with LN biopsy	39	25
Negative	18 (46.2)	9 (36)
Positive	21 (53.8)	16 (64)
Metastasis		
Absent	11 (21.6)	4 (7.4)
Present	40 (78.4)	50 (92.6)
Number of metastatic sites		
≤mean (≤3)	34 (66.7)	31 (57.4)
>mean (>3)	17 (33.3)	23 (42.6)
FIGO staging		
Early (Stage I & II)	11 (21.6)	4 (7.4)
Advanced (Stage III & IV)	40 (78.4)	50 (92.6)
Histological Subtype		
LGSOC	35 (68.6)	4 (7.4)
HGSOC	16 (31.4)	50 (92.6)
Uterine body involvement		
Absent	32 (62.7)	30 (55.6)
Present	19 (37.3)	24 (44.4)
Fallopian tube epithelial involvement		
Absent	47 (92.2)	49 (90.7)
Present	4 (7.8)	5 (9.3)
Associated non-invasive component (Borderline Tumour)		
Absent	26 (51)	34 (63)
Present	25 (49)	20 (37)
Psammoma bodies		
Absent or focal	31 (60.7)	39 (72.2)
Diffuse	20 (39.2)	15 (27.8)
Necrosis		
Absent	41 (80.4)	39 (70.4)
Present	10 (19.6)	16 (29.6)

**Table 3:** Multivariate Cox proportional hazards regression model analysis of disease-free survival of serous ovarian carcinoma patients in the validation set.

Factor	Coefficient	Hazard Ratio (HR)	95% Confidence Interval (CI)	*p*-Value
Low TSR	0.6428	1.9019	(0.7854, 4.6054)	0.1543
Mature Stromal Type	−0.8968	0.4079	(0.1818, 0.9149)	0.0296
Low Peritumoural TILs	0.8886	2.4317	(0.9645, 6.1305)	0.0596

## Data Availability

The datasets used and/or analysed during the current study are available from the corresponding author upon reasonable request.

## Supplementary Materials

The following supporting information can be downloaded at: https://www.mdpi.com/article/10.3390/cancers16213611/s1, Table S1: Multivariate cox proportional hazards regression model analysis of Disease-free survival of serous ovarian carcinoma patients in the discovery set; Table S2: Summary of key histopathological Features and their diagnostic and prognostic relevance in Low-Grade and High-Grade Serous Ovarian Carcinoma (LGSOC and HGSOC).
