# Supplementary material for: Serous Ovarian Carcinoma: Detailed Analysis of Clinico-Pathological Characteristics as Prognostic Factors
Source: Cancers (Basel). 2024 Oct 25;16(21):3611. doi: 10.3390/cancers16213611 (PMC11545192; doi:10.3390/cancers16213611)
Supplement: Supplementary file 1 [file cancers-16-03611-s001.zip › cancers-3258949-supplementary.pdf]

Table S1. Multivariate cox proportional hazards regression model analysis of Disease-free survival of serous ovarian carcinoma patients in the discovery set.

Factor	Coefficient	Hazard Ratio (HR)	95% Confidence Interval (CI)	p- value
**High Peritumoural Tumour Budding (PTB)**	0.5430	1.205	(0.8726, 2.632)	0.191
**Low Tumour-stroma Ratio (TSR)**	0.7642	1.637	(0.8716, 1.9227)	0.0962
**Mature Stromal Type**	- 0.4628	0.389	(0.1645, 0.8760)	**0.002**

Table S2: Summary of key histopathological Features and their diagnostic and prognostic relevance in Low-Grade and High-Grade Serous Ovarian Carcinoma (LGSOC and HGSOC)

**Histopathological Feature**	**Diagnostic and Prognostic Relevance**
**LVSI (Lymphovascular Space Invasion)**	Indicator of aggressive behaviour and lymph node metastasis.<br>Strongly associated with **HGSOC**, less commonly observed in **LGSOC**.
**MVD (Microvessel Density)**	Linked to tumour aggressiveness and metastatic potential.<br>Higher in **HGSOC**, while typically lower in **LGSOC**.
**TB (Tumor Budding)**	Indicator of metastatic potential and poorer outcomes.<br>Frequently seen in **HGSOC** but rare or absent in **LGSOC**.
**TILs (Tumor-Infiltrating Lymphocytes)**	Predictor of better prognosis and therapy responses.<br>**High peritumoral TILs** are associated with **HGSOC**, while **low TILs** are more typical in **LGSOC**.
**TSR (Tumor-Stroma Ratio)**	Prognostic predictor of disease-free survival.<br>**Low TSR** (more stroma) is linked with **HGSOC**, while **high TSR** (less stroma) is often seen in **LGSOC**.
**Stromal Type**	Independent prognostic marker for disease-free survival.<br>**Immature-intermediate stroma** is common in **HGSOC**, while **mature stroma** is more frequent in **LGSOC**.
**TLS (Tertiary Lymphoid Structures)**	Associated with better overall survival.<br>More commonly found in **HGSOC**, while rare in **LGSOC**.
